# Proximal femoral fixation method and axial load affect simulated muscle forces in an ex vivo feline limb press

**DOI:** 10.1111/vsu.14252

**Published:** 2025-04-07

**Authors:** Parisa Mazdarani, Michelle B. M. Nielsen, James E. Miles

**Affiliations:** ^1^ Department of Veterinary Clinical Sciences University of Copenhagen Copenhagen Denmark; ^2^ Present address: College of Veterinary Medicine University of Florida Gainesville Florida USA

## Abstract

**Objective:**

To quantify how increasing axial loads combined with different femoral fixation methods impact simulated quadriceps and gastrocnemius muscle forces.

**Study design:**

Experimental, non‐randomized, ex vivo study.

**Methods:**

A custom limb press permitting axial loads of 10%–40% bodyweight with three femoral fixation models was tested with 24 limbs from 12 cats (4 per method). Fixation models were: one–complete hip mobility, two–rigid femoral fixation, three–flexion‐extension hip mobility. Femoral angulation to horizontal of 60° ±5° and stifle/hock angulations of 120° ±5° were maintained using turnbuckles. Primary outcomes were bodyweight normalized quadriceps and gastrocnemius forces, and their ratio. Secondary outcomes were radiographic limb angulation and relative foot position for models 2 and 3.

**Results:**

Normalized quadriceps forces increased more with axial load in models 1 and 3 than model 2 (*p* = .04), whereas normalized gastrocnemius force increased more with model 2 than models 1 and 3 (*p* = .009). Force ratios were unaffected by axial load (*p* = .4), but model 2 ratios were lower than models 1 and 3 (*p* = .007). Femoral angulation did not differ with load (*p* = .1) or model (*p* = .9), but both stifle and hock flexed with increasing load (*p* < .001) but remained largely within target. Relative foot position was mean 3.9 mm more caudal in model 2 than model 3.

**Conclusion:**

Simulated muscle forces were proportionate to axial load, while rigid femoral fixation negatively affected both forces and ratios.

**Clinical significance:**

Retaining hip flexion‐extension yields normalized quadriceps forces closer to in vivo values and could improve current ex vivo models.

## 1 INTRODUCTION

Limb press models permit ex vivo investigations of the stifle joint at chosen angulations, under axial loads and with a limited number of simulated muscle loads, enabling testing of selected surgical procedures. Results from these models are frequently used to justify and inform surgical techniques for stabilization of the stifle joint. Ex vivo models are one method of achieving the 3R goals of replacement, reduction and refinement in preclinical research,^1^ but models must be physiologically valid if these 3R aims are to be realized. Ex vivo assessments of cranial cruciate ligament surgeries using feline limb press models adapted from canine studies include tibial tuberosity advancement,^2^ tibial plateau leveling osteotomy,^3^ intracapsular stabilization,^4^ and extracapsular stabilization.^4^, ^5^, ^6^ Neither of the osteotomy techniques provided stability to the cranial cruciate ligament‐deficient feline stifle using these limb press models,^2^, ^3^ in contrast to similar models in the dog.^7^, ^8^, ^9^, ^10^, ^11^ These ex vivo feline studies have used a rigidly fixed proximal femur, replacement of the quadriceps and gastrocnemius muscles with wires and turnbuckles approximating their anatomical position, and subsequent induction of simulated muscle loads by application of an axial load.

One feline limb press study reported mean simulated quadriceps loads of 19.8 N (SD 5.7 N) and 16.5 N (SD 4.9 N) with axial loads of 30% and 10%, respectively, of bodyweight in cats with a mean body mass of 3.7 kg (SD 1.1 kg). See Suppl. S17 in Ref. 5. In vivo patellar ligament loads of approximately 80 N and 50–60 N have been reported for walking cats during the early and mid‐late stance phases.^12^ Simulated gastrocnemius loads or the ratio of quadriceps to gastrocnemius loads have not been reported for tested feline models, but forces of 27 N have been reported for walking cats,^12^, ^13^ while patellar ligament to gastrocnemius force ratios of approximately 2–3:1 during early to mid‐stance may be estimated from Hasler et al.^12^ Increased stability with increased quadriceps loads has been demonstrated in a canine tibial tuberosity advancement model,^14^ and preactivation of the quadriceps in a closed kinetic chain situation has been shown to limit cranial tibial translation in an ex vivo model.^15^ The wide disparity between reported physiologic loads and ratios compared to those achieved in published limb press models could be a potential explanation for the failure to achieve ex vivo stability for osteotomy‐based stifle stabilization in the cat.^2^, ^3^ This failure is at odds with apparent good clinical success for these procedures,^16^, ^17^, ^18^, ^19^, ^20^ and understanding simulated muscle load magnitudes in these models may help improve limb press validity and guide development of technique variations appropriate to cats, with obvious benefits for affected animals and treating clinicians.

The objective of this study was to identify changes in simulated muscle loads in feline limb press models with varying axial loads and different proximal femoral fixation methods. We hypothesized that rigid femoral fixation would result in lower‐than‐expected simulated quadriceps loads, and that permitting hip mobility within the limb press model would induce simulated muscle loads approximating physiologic loading of the feline stifle joint with bodyweight normalized quadriceps and gastrocnemius forces approximately 1.8 and 0.64,^12^ respectively.

## 2 MATERIALS AND METHODS

Approval for this study was obtained from the local ethical administrative committee (no. 2022‐16). Client‐owned cat cadavers euthanized for reasons other than this study and with written permission for research or teaching use were used. Exclusion criteria were evidence of orthopedic disease or skeletal immaturity identified on palpation or radiographic screening.

### 2.1 Limb press design

A custom limb press inspired by previous reports^2^, ^3^, ^4^, ^5^, ^6^ was constructed from commonly available materials (Data S1). The top plate permitted three proximal femoral fixation methods (one–complete hip mobility, two–rigid femoral fixation, three–flexion‐extension hip mobility).

### 2.2 Load cell preparation

Two DYMH‐103 load cells with a rated load of 0–10 kg and overload tolerance of 150% rated maximum load were wired to HX711 amplifiers (SparkFun, Niwot, Colorado) and a DFRduino UNO R3 board (DFRobot, Shanghai, China). Publicly available code was used for calibration and monitoring.^21^, ^22^ Calibration was repeated on each experimental day and controlled at each end of activities.

Each load cell was sequentially loaded and unloaded using weights from 0 N to 73.7 N to test linearity and repeatability against the manufacturer's specifications (Table S1).

### 2.3 Limb preparation

Each cadaver was weighed prior to harvesting. Eight limbs from four cats were used for each model (12 cats total). Limbs were harvested by partial hemipelvectomy (model 1) or coxofemoral disarticulation (models 2 and 3), and frozen until use at −18°C, defrosting for 12–24 h at 5°C beforehand. Soft tissues were removed while preserving the joint envelopes at the coxofemoral (model 1 only), femorotibial and tibiotarsal joints.

A craniocaudal tunnel was drilled in the mid‐proximal patella and either a screw eye hook (model 1) or a bent 4.5 mm reconstruction plate (with two 3.5 mm cortical screws) was attached to the proximal femur for application of the simulated quadriceps load. Two 2 mm cortical bone screws were placed proximally at the medial and lateral femorofabellar articulations and a mediolateral tunnel was drilled in the proximal calcaneus for application of the simulated gastrocnemius load. Joint tissues were kept moist as needed using isotonic saline.

The simulated muscle linkages consisted of a calibrated and zeroed load cell in series with a turnbuckle, attached to the bone tunnels using cerclage wire or suture material and to the screw eye hook (model 1) or plate (models 2 and 3) with a threaded nut.

### 2.4 Model 1–complete hip mobility

**FIGURE 1:**
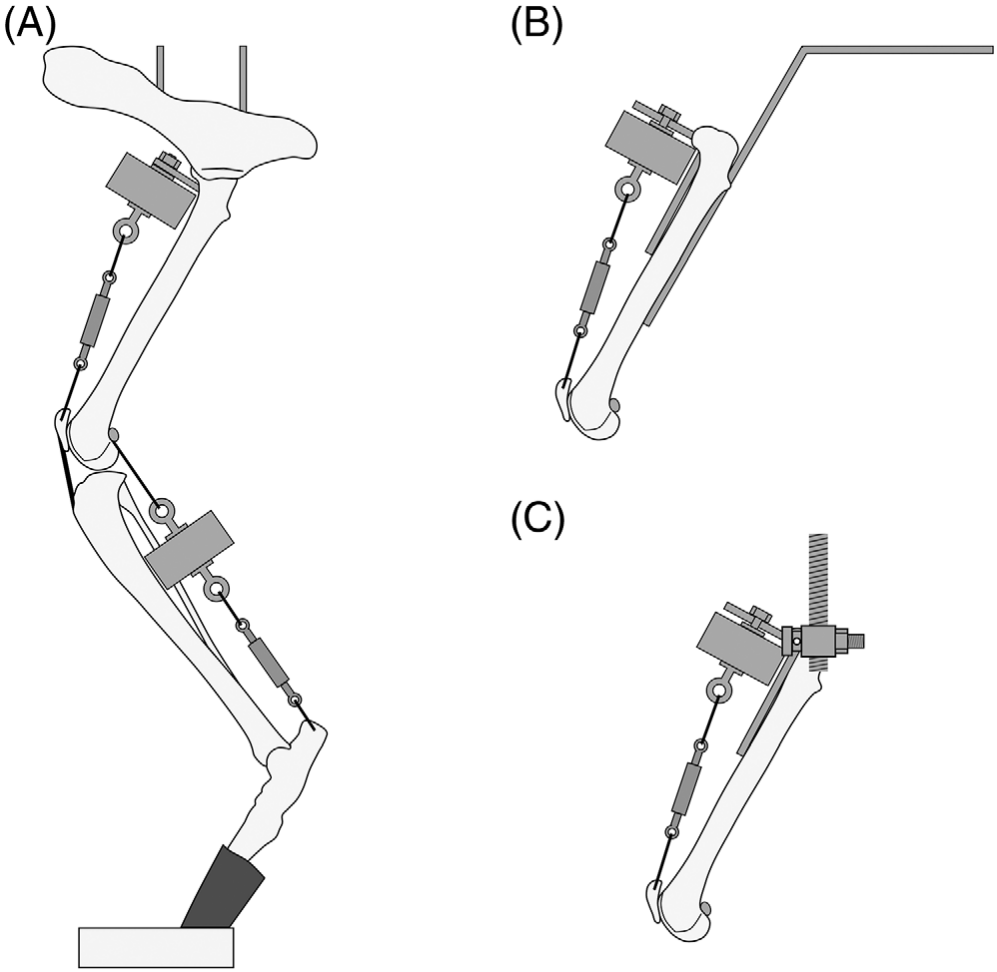
Limb mounting details. (A) Hemipelvectomy model (model 1) with intact hip joint, supported by two positive‐profile pins attached to the top plate. The foot is positioned in a receiving cup on top of a digital scale (not shown). The quadriceps load cell is attached proximally to a screw eye hook, and via a turnbuckle to the patella. The gastrocnemius load cell is attached to screws placed at the approximate locations of the fabellae and via a turnbuckle to a tunnel in the caudoproximal calcaneus. (B) Fixed femur model (model 2), with femur stabilized using bent 3.5 mm dynamic compression plate attached to the caudal aspect of the femur and top plate. The quadriceps load cell is attached to a bent 4.5 mm reconstruction plate attached to the cranial aspect of the femur. (C) Constrained femur model (model 3), with femur supported by a mediolateral transverse pin through the femoral head, which passes through medial and lateral external fixator clamps. The quadriceps load cell is attached as for (B).

**FIGURE 2:**
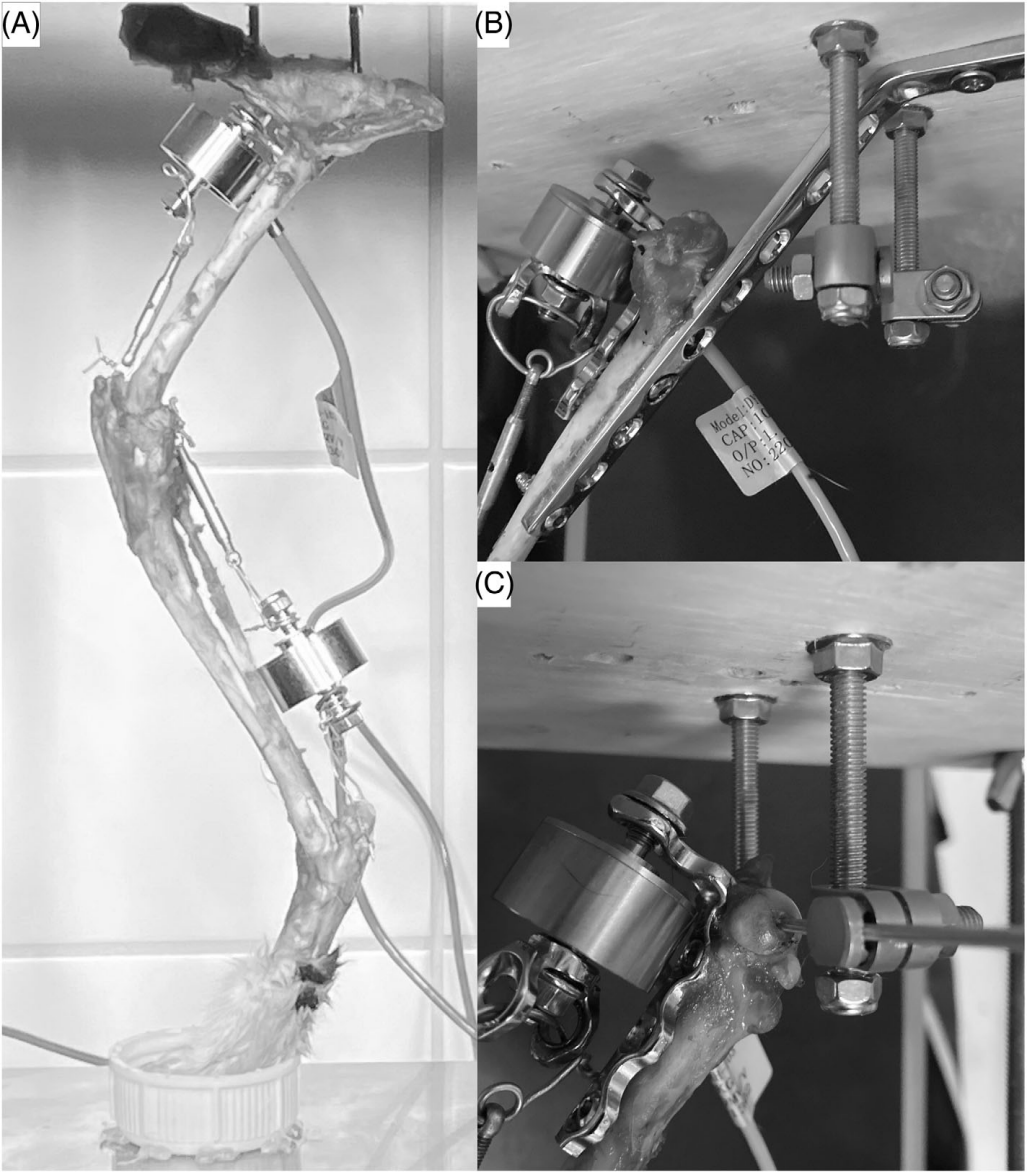
Experimental set up. (A) Hemipelvectomy model, (B) detail of attachment for fixed femur model and (C) detail of attachment for constrained femur model.

Limbs were mounted in the press using two 2.0/2.4 mm positive‐profile external fixation pins driven vertically into the ilium and ischium adjacent to the acetabulum (Figures 1A and 2A).

Using the top plate, compression was applied to achieve a registered load of 10% of body mass on the digital scale. The femorotibial and tibiotarsal angles were adjusted to 120° ±5° using the turnbuckles and custom templates aligned with the greater trochanter, the lateral collateral ligament of the femorotibial joint, the lateral malleolus, and the distal metatarsus. Femoral angle was adjusted to 60° ±5° by moving the foot craniocaudally and confirmed using a custom template aligned with the top plate, greater trochanter and the lateral collateral ligament. Angulation was not confirmed radiographically, due to rotation of the limb. Axial loading was increased in 10% steps to 40% body mass and simulated quadriceps and gastrocnemius loads were read from the load cells once a stable axial load was achieved and following confirmation of limb positioning and joint angles.

### 2.5 Model 2–rigid femoral fixation

A bent 3.5 mm dynamic compression plate was applied to the caudal aspect of the femur and attached to the top plate to produce a femoral angulation of 60° (Figures 1B and 2B).

Using the top plate, compression was applied to achieve axial loads of 10%–40% of body mass on the digital scale, after initially overloading and unloading the construct to confirm security of instrumentation and remove slack from suture or cerclage fixation. Satisfactory limb angulation was confirmed using radiography, aiming for superimposition of the femoral condyles to within 1 mm.^4^, ^5^ Simulated quadriceps and gastrocnemius loads were read from the load cells at each axial load.

### 2.6 Model 3–flexion‐extension hip mobility

A 2 mm tunnel was drilled through the femoral head, from the fovea capitis to the greater trochanter, using a pin aligned with the caudal aspects of the femoral condyles as a visual guide. Limbs were mounted in the press using a 2 mm pin placed through this tunnel and attached to external fixator clamps on two 5 mm threaded rods (Figures 1C and 2C).

Using the top plate, compression was applied to achieve a registered load of 10%–40% of body mass on the digital scale, after initially overloading and unloading the construct. The femorotibial and tibiotarsal angles were adjusted to 120° ±5° using the turnbuckles, while the femoral angle was adjusted to 60° ±5° by moving the foot cranially or caudally prior to commencing the measurement process. Satisfactory angulation and positioning were confirmed using radiography, as before. Simulated quadriceps and gastrocnemius loads were read from the load cells.

### 2.7 Radiographic measurements

The x‐coordinates for the center of the femoral head, mid‐point of Blumensaat's line, center of the talus, and distal metatarsus were measured for all images for models 2 and 3. Relative foot position was expressed as the ratio of the distance of the distal metatarsus from the mid‐point of Blumensaat's line compared to the distance from the mid‐point of Blumensaat's line to either the center of the femoral head (FS) or the center of the talus (SH).

Repeat measurements of limb angulation were performed at the completion of data collection for models 2 and 3.

### 2.8 Statistical analysis

Load cell data were normalized to bodyweight. Statistical analyses were performed using SPSS 28 (IBM Corp, Armonk, New Jersey). A mixed‐model repeated‐measures ANOVA was used to explore the primary outcomes with model as a between‐subjects effect, and limb and axial load as within‐subject effects, including their interactions, such that: $\text{mean}\;\left(Y_{\mathrm{ijk}}\right)=\text{intercept}+{\text{limb}}_{i}+{\text{model}}_{j}+{\text{load}}_{k}+{\left(\text{limb}\times \text{model}\right)}_{\mathrm{ij}}+{\left(\text{limb}\times \text{load}\right)}_{\mathrm{ik}}+{\left(\text{model}\times \text{load}\right)}_{\mathrm{jk}}+{\left(\text{limb}\times \text{model}\times \text{load}\right)}_{\mathrm{ijk}}$, where Y represents either of the normalized muscle forces or their ratio, i the levels of limb (left, right), j the levels of model (1–3), and k the levels of axial load (10%, 20%, 30%, and 40%).

Residuals were assessed for normality using the Shapiro–Wilk test and quantile‐quantile plots. Effects sizes were estimated using generalized eta‐squared ($\eta_{G}^{2}$), with cutoffs between small, medium and large effects at 0.06 and 0.14.^23^ Femoral and joint angulations, as well as the two measures of relative foot position were explored similarly.

Significance was set at the 5% level.

## 3 RESULTS

Mean body masses were 3.0 kg (SD 0.5 kg), 4.6 kg (SD 0.9 kg), and 5.6 kg (SD 1.2 kg) for models 1, 2, and 3, respectively.

### 3.1 Specification check

Compared to the manufacturer's specification of 0.30% of maximum rated load for non‐linearity, we measured errors of 0.06%–0.85% and 0.05%–0.35% for the two load cells. By using the worst rated load cell for the gastrocnemius, maximum absolute error magnitudes of 0.20–0.4 N could be expected across the predicted range of both simulated muscle forces.

Maximum hysteresis for paired measurements in increasing and decreasing loading cycles were 0.07% and 0.06% for the two load cells compared to a listed specification of 0.03% of maximum rated load.

### 3.2 Normalized quadriceps force

**FIGURE 3:**
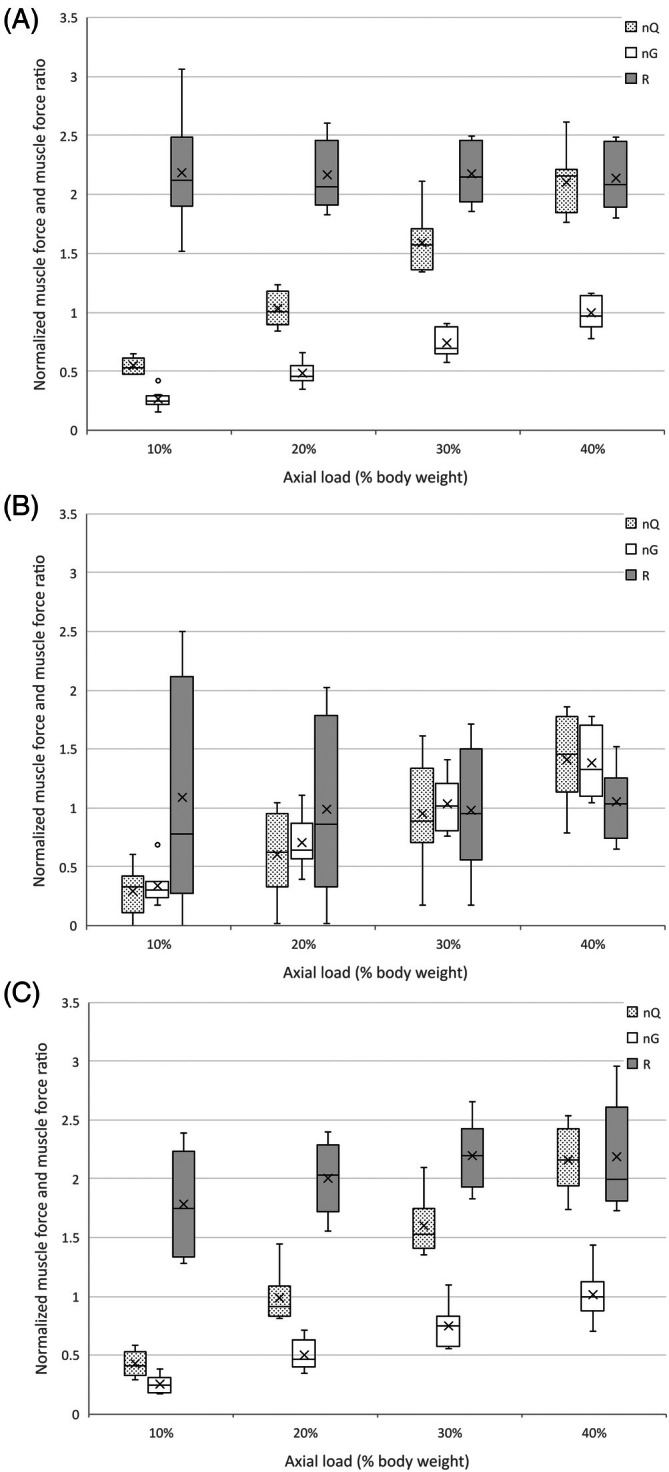
Box and whisker chart of normalized quadriceps and gastrocnemius forces and force ratios at axial loads of 10%–40% bodyweight. (A) intact hip joint (model 1), (B) rigid femoral fixation (model 2), (C) flexion‐extension femoral mobility (model 3).

Normalized quadriceps force reached likely physiologic levels between 30% and 40% axial loads in models 1 and 3 but remained subphysiologic in model two at 40% (Table 1, Figure 3). Mauchly's test indicated the assumption of sphericity was violated for axial load (*χ* ^2^_(5)_ = 11.7, *p* = .04) and Greenhouse–Geisser corrections are reported for this effect (*ε* = 0.56). Normalized quadriceps force increased with increasing axial load (*F*_(2,1.7)_ = 405.57, *p* < .001, $\eta_{G}^{2}$ = 0.85) and varied with model (*F*_(2,9)_ = 7.22, *p* = .01, $\eta_{G}^{2}$ = 0.62), with the interaction model × load demonstrating a lower response for model two than for models 1 and 3 with increasing axial load (*F*_(2,3.3)_ = 6.67, *p* = .04, $\eta_{G}^{2}$ = 0.16). Values for model 2 at 10% axial load were lower than model 1 (*p* = .04), and at 30% and 40% axial loads lower than both models 1 (*p* = .03, *p* = .03) and 3 (*p* = .02, *p* = .01). No other interaction effects were observed (Table S2).

**TABLE 1:** Mean raw simulated muscle forces in newtons at axial loads of 10%–40% of bodyweight, along with means of values normalized to bodyweight used for statistical analysis.

Model	Muscle	10%	20%	30%	40%
1	Quadriceps	15.8 (SD 3.4)	30.1 (SD 8.2)	46.1 (SD 10.3)	60.9 (SD 12.8)
	Gastrocnemius	7.7 (SD 3.2)	14.1 (SD 4.3)	21.4 (SD 5.3)	28.7 (SD 6.3)
	nQ	0.55 (SD 0.07)	1.03 (SD 0.15)	1.59 (SD 0.26)	2.1 (SD 0.27)
	nG	0.26 (SD 0.08)	0.48 (SD 0.09)	0.74 (SD 0.12)	0.99 (SD 0.14)
2	Quadriceps	13 (SD 9.9)	27.7 (SD 19.1)	44.1 (SD 25.1)	64.6 (SD 25.1)
	Gastrocnemius	14.7 (SD 5.5)	31.3 (SD 9.9)	46 (SD 11.7)	61.7 (SD 17.4)
	nQ	0.29 (SD 0.2)	0.6 (SD 0.37)	0.96 (SD 0.45)	1.41 (SD 0.38)
	nG	0.34 (SD 0.16)	0.7 (SD 0.22)	1.03 (SD 0.22)	1.38 (SD 0.29)
3	Quadriceps	23.7 (SD 8.3)	54.5 (SD 18.8)	88 (SD 24.6)	117.8 (SD 26.3)
	Gastrocnemius	13.9 (SD 6)	28 (SD 11.3)	41.6 (SD 15.7)	56.5 (SD 20.1)
	nQ	0.43 (SD 0.1)	0.98 (SD 0.21)	1.6 (SD 0.24)	2.16 (SD 0.27)
	nG	0.25 (SD 0.07)	0.5 (SD 0.13)	0.74 (SD 0.18)	1.02 (SD 0.22)

Abbreviations: Model 1, model with intact hip joint; Model 2, model with femur fixed at 60°; Model 3, model with mediolateral pin through femoral head; nG, bodyweight normalized gastrocnemius force; nQ, bodyweight normalized quadriceps force; SD, standard deviation.

### 3.3 Normalized gastrocnemius force

Normalized gastrocnemius force reached likely physiologic levels between 20% and 30% axial loads in models 1 and 3, and between 10% and 20% axial loads in model 2 (Table 1, Figure 3). Mauchly's test indicated the assumption of sphericity was violated for axial load (*χ* ^2^_(5)_ = 26.1, *p* < .001) and Greenhouse–Geisser corrections are reported for this effect (*ε* = 0.40). Normalized quadriceps force increased with increasing axial load (*F*_(2,1.2)_ = 531.01, *p* < .001, $\eta_{G}^{2}$ = 0.85) and varied with model (*F*_(2,9)_ = 9.14, *p* = .007, $\eta_{G}^{2}$ = 0.42), with the interaction model × load demonstrating a greater response for model two than for models 1 and 3 with increasing axial load (*F*_(2,2.4)_ = 7.16, *p* = 0.009, $\eta_{G}^{2}$ = 0.13). Values for model 2 at 20%, 30%, and 40% axial loads were greater than both models 1 (*p* = .02, *p* = .005, *p* = .02, respectively) and 3 (*p* = .03, *p* = .005, *p* = .02, respectively). No other interaction effects were observed (Table S2).

### 3.4 Muscle force ratios

Mauchly's test indicated the assumption of sphericity was violated for axial load (*χ* ^2^_(5)_ = 12.5, *p* = .03) and Greenhouse–Geisser corrections are reported for this effect (*ε* = 0.52). Ratios were unaffected by increasing axial load (*F*_(2,1.6)_ = 0.83, *p* = .4, $\eta_{G}^{2}$ = 0.02) but were lower for model 2 than models 1 and 3 (*F*_(2,9)_ = 12.5, *p* = .007, $\eta_{G}^{2}$ = 0.72). Pairwise comparison showed that model 2 values were lower than model 1 at all axial loads (*p* = .02, *p* < .001, *p* < .001, *p* < .001, respectively) and lower than model 3 at axial loads of 20%, 30%, and 40% (*p* < .001 for each).

Limb side (*F*_(1,2)_ = 13.6, *p* = .005, $\eta_{G}^{2}$ = 0.21), limb × model (*F*_(2)_ = 18.48, *p* < .001, $\eta_{G}^{2}$ = 0.42), and load × limb × model (*F*_(2,3.4)_ = 5.22, *p* = .009, $\eta_{G}^{2}$ = 0.12) affected calculated ratios, with right limb values exceeding left limb values in model 1 (*p* = .01) and 2 (*p* < .001), and lower than left limb values in model 3 (*p* = .03). For model 1, this was observed at axial loads of 20%, 30%, and 40%, for model 2 at all axial loads, and for model 3 only at axial loads of 10% and 20%. No other interaction effects were observed (Table S2).

### 3.5 Radiographic limb angulation (models 2 and 3)

No violations of sphericity were observed for femoral, stifle or hock angulation.

Femoral angulation was unaffected by axial load (*F*_(2,3)_ = 2.45, *p* = .1, $\eta_{G}^{2}$ = 0.08) or model (*F*_(1,6)_ = 0.02, *p* = .9, $\eta_{G}^{2}$ = 0.001), and no other interaction effects were noted (Table S3).

**FIGURE 4:**
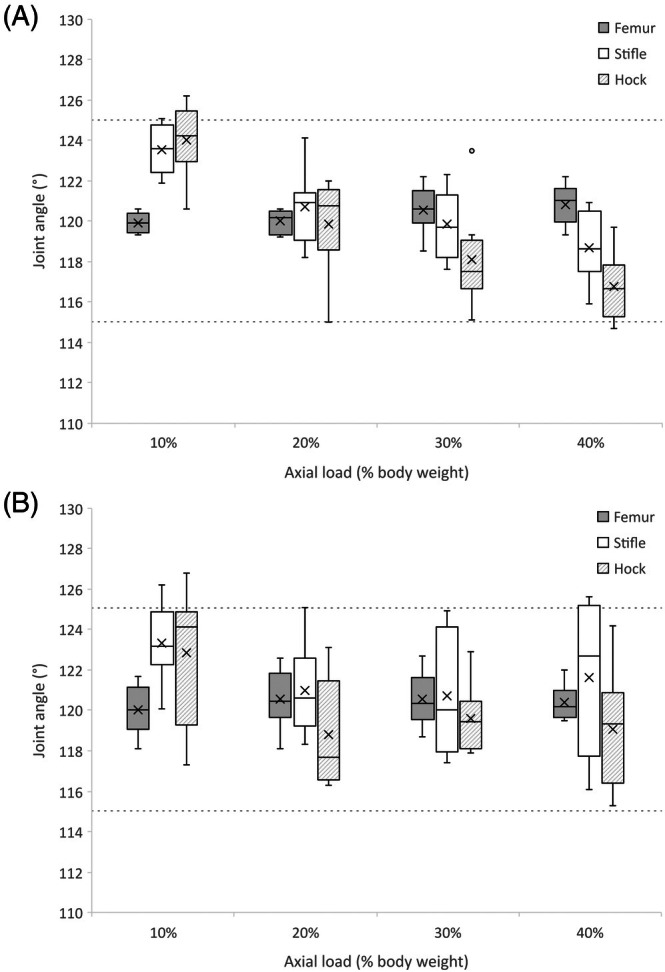
Box and whisker chart of limb angles for models 2 and 3 under axial loads of 10%–40% bodyweight. For clarity, the femoral angle is reported as the caudal angle between a horizontal line passing through the greater trochanter and progressing anticlockwise towards the stifle joint. The stifle and hock angles represent caudal and cranial joint angles, respectively. Target ranges for the stifle and hock angles are indicated with dotted lines. (A) fixed femur model (model 2), (B) flexion‐extension femur model (model 3).

Although caudal stifle joint angulation remained within target ranges for the majority of limbs (Figure 4) and was unaffected by model (*F*_(1,6)_ = 1.81, *p* = .2, $\eta_{G}^{2}$ = 0.05), flexion with increasing axial load occurred (*F*_(2,3)_ = 10.06, *p* < .001, $\eta_{G}^{2}$ = 0.30). Caudal joint angle at axial loads of 10% exceeded those at 20%, 30%, and 40% (*p* = .03, *p* = .02, *p* = .01, respectively), but the remaining stepwise load increases had no effect. No other interaction effects were observed (Table S3).

Similarly, cranial hock joint angulation remained within target ranges for the majority of limbs (Figure 4) and was unaffected by model (*F*_(1,6)_ = 0.28, *p* = .6, $\eta_{G}^{2}$ = 0.01), but flexion with increasing axial load was observed (*F*_(2,3)_ = 14.21, *p* < .001, $\eta_{G}^{2}$ = 0.50). Values at axial loads of 30% and 40% were lower than at 10% (*p* = .01, *p* = .002, respectively), but no stepwise differences were observed. No other interaction effects were observed (Table S3).

### 3.6 Relative foot position (models 2 and 3)

No violations of sphericity were observed for either foot position ratio.

Relative foot position FS was unaffected by either model (*F*_(1,6)_ = 4.76, *p* = .07, $\eta_{G}^{2}$ = 0.17) or axial load (*F*_(2,3)_ = 1.74, *p* = .2, $\eta_{G}^{2}$ = 0.04) or any other terms. In contrast, relative foot position SH was on average 6.7% (95% CI: 0.7%; 12.7%) more caudal in model 2 than in model 3 limbs (*F*_(1,6)_ = 7.53, *p* = .03, $\eta_{G}^{2}$ = 0.26) but was unaffected by axial load (*F*_(2,3)_ = 0.84, *p* = .5, $\eta_{G}^{2}$ = 0.01) or any other terms. Based on grand mean stifle joint‐hock joint distances across both models and all axial loads, this corresponded to a mean 3.9 mm (95% CI: 0.4 mm; 7.4 mm) difference in foot positioning between models 2 and 3. No other interaction effects were observed (Table S4).

## 4 DISCUSSION

We demonstrated a clear relationship between normalized simulated muscle loads and the applied axial load in a limb press for all tested models. Normalized quadriceps forces approximated patellar ligament forces measured in vivo at higher axial loads in models 1 and 3 but not model 2. Muscle force ratios were model‐dependent and relatively consistent for models 1 and 3, which approached in vivo values, whereas model 2 produced widely variable muscle force ratios, which could cast doubt on results from current models. Constraining the hip to flexion‐extension (model 3) appears to preserve the benefits of complete hip freedom of movement while facilitating experimental use and radiographic documentation and may be beneficial for future investigations.

Previously reported feline patellar ligament loads in a limb press model similar to our model 2 appear to be markedly below physiologic loads, and consistent with a subset of our data.^5^, ^12^ This model is characterized by a rigidly fixed proximal femur^2^, ^3^, ^4^, ^5^, ^6^: the quadriceps force generated by axial loading can be expected to be relatively limited due to the proximity of the line of action from the ground reaction force to the stifle joint center of rotation. The marked variability in muscle force ratios particularly at lower axial loads in our data may result from the similar values and low magnitudes of the quadriceps and gastrocnemius forces at these loads. Mathematically, small changes in either the numerator or denominator of the ratio could thus result in marked fluctuations in calculated ratios. Permitting flexion and extension at the level of the hip, as in our models 1 and 3, results in an additional torque with a longer moment arm originating from the femoral head. As a result, much larger quadriceps forces are required for maintaining the selected stifle joint angle, and the ratio of quadriceps and gastrocnemius forces approached those reported in vivo.^12^

Whether near‐physiologic muscle loads or their ratio is the main factor for improved validity in ex vivo models remains unclear. While an axial load of 30% bodyweight has been used in previous studies, our data suggest that the exact percentage may not be critical if the key consideration is force ratio, rather than values for the quadriceps or gastrocnemius forces. However, joint stability may additionally depend on other factors which could be influenced by increasing axial load, such as tension in the joint capsule, surrounding ligaments, and menisci, which were not evaluated in this study.

Quadriceps forces achieved in model 1 did not reach the peak patellar ligament forces reported by Hasler et al.^12^ This may reflect that the patellar ligament forces only represent data from one cat from a sample population with masses of 3.8–5.0 kg, somewhat heavier than those used in this model. We did not measure patellar ligament forces, which may not be equivalent with quadriceps forces, given that a lever‐arm effect at the patella has been reported in humans.^24^ However, the larger cadavers used for model 3 produced values in good agreement with this earlier report, and our statistical comparisons were based on bodyweight normalized forces which should remove the effect of varying cat size. Force ratios in models 1 and 3, while likely superior to those achieved in earlier feline limb press models, and better than those found in model 2, remained subphysiologic compared to most of the stance phase. This appears to be due to the higher gastrocnemius forces relative to the quadriceps forces developed in models with rigidly fixed proximal femora. One explanation for this is that unlike the dog, the cat has a soleus muscle, with muscle fibers specialized for postural recruitment.^25^ Whereas the gastrocnemius muscle is biarticular, the soleus muscle arises from the head of the fibula and is uniarticular.^26^ A recent experimental study yielded a peak medial gastrocnemius force of ca. 27 N and peak soleus force of ca. 5 N from cats with body mass of 5.2 kg (SD 1.1 kg) during level walking.^13^ The limb press methods required to simulate this are not clear. Other potential issues include choice of attachment points for the simulated muscles, which could influence their lines of action and subsequent moment arms. The cause of force ratio variation with limb side is unclear given the lack of effect on normalized quadriceps and gastrocnemius forces, but could reflect unconscious bias in instrumentation, setting joint angles or limb positioning between left and right limbs.

While both models 1 and 3 appear quantitatively superior to model 2 in terms of achieved forces and ratios, model 3 was qualitatively superior for research use. During changing axial loading, varying degrees of internal and external rotation were induced at the hip joint in model 1. This made radiographic documentation impossible within the time and financial constraints we had for experimental work, since multiple exposures would have been required to achieve femoral condylar superimposition each time. Joint and femoral angles were set in similar fashion to the other two models, and were likely similar, despite the inability to subsequently confirm them. Constraining the foot might have simultaneously constrained limb rotation but was not attempted. In contrast, model 3, which limited femoral movement to flexion/extension appeared to give the same benefits in terms of loading while simplifying experimental use. The limb angulations used in this study were based on previous reports in cats^2^, ^3^, ^4^, ^5^, ^6^ which have been claimed to be consistent with the early stance phase. However, joint angle data from walking cats indicates that a slightly more flexed stifle and hock position may be more representative of the early stance phase,^12^, ^27^ with the selected angles more consistent with mid‐ to late‐stance. Despite consistent efforts with goniometry and concomitant radiographical measurements, subsequent analysis demonstrated that not all limbs achieved target angulations. Whether these disparities affected the presented results is unclear. The small difference in relative foot position between models 2 and 3 was unexpected, given that foot position was set similarly based on femoral, stifle and hock joint angles in each. Caudal positioning should theoretically increase simulated quadriceps force and could explain the slightly higher quadriceps forces we report compared with a previous study. See Suppl. S17 in Ref. 5.

Tibial tuberosity transposition beyond a patella tendon angle of 90° failed to restore stifle stability in cat hindlimbs with a rigidly fixed femur and subjected to a 30% bodyweight axial load.^2^ The imbalance seen in model 2 might help explain this poor stability since the premise of this osteotomy is that reorienting the quadriceps force will result in joint stability^28^ and because increased quadriceps force correlates with increasing joint stability in the dog.^14^, ^15^ Likewise, tibial plateau leveling osteotomy under similar conditions failed to restore stifle joint stability even at tibial plateau angles of 0° and −5°.^3^ This is more challenging to explain, since the primary aim of this technique is neutralization of cranial tibial thrust.^29^ We did not specifically test these situations, and testing feline osteotomies under higher quadriceps loads would be instructive. Other biomechanical or anatomical factors may be responsible. These could include tibial plateau topography, and limitation of the model to only two muscles, ignoring the contributions of the hamstrings.^30^

Whether similar issues occur in fixed‐femur canine limb press models is uncertain, although one study reported mean patellar ligament loads of 1.4–4.6 N^8^ suggesting similar issues might exist. Canine limb press models have tended to show consistent stability in cranial cruciate ligament‐deficient stifles managed with osteotomy techniques,^8^, ^9^, ^10^, ^11^, ^31^ in stark contrast to in vivo data,^32^, ^33^, ^34^, ^35^, ^36^ and results from these models remain controversial. Despite this, clinical outcomes after canine osteotomy techniques are considered to be good.^37^ Since similar in vivo data for osteotomy procedures in cats is lacking, it remains unclear whether the current feline models in the literature accurately reflect in vivo results or if they underestimate clinical stability, or if use of a model with more physiologically accurate loads would produce different outcomes. However, it would be sensible to test feline stifle stabilization methods in a model which more accurately reflects physiologic forces than current models appear to, if surgeons are to be guided as to the applicability (or otherwise) of these methods in clinical practice. Whether limb press models can be useful remains to be determined. Canine limb press models using hemipelvectomized hindlimbs have yielded muscle force ratios similar to those found here,^38^, ^39^ while alternative canine models exploring triple tibial osteotomy and CORA‐based leveling osteotomy which focused on controlling muscle force ratio rather than axial loading have produced evidence of joint instability which might better reflect in vivo performance.^30^, ^40^ Critical evaluation of canine limb press models appears warranted.

Simulated muscle forces in this limb press varied with both axial load and femoral constraint. All tested models remain limited by simulation of only two muscle groups, although permitting femoral movement appears to produce simulated muscle loads and ratios closer to expected values than currently described models, from which results may be unreliable. Choice of axial load and femoral fixation could influence the reliability of studies investigating stifle stabilization techniques.

## AUTHOR CONTRIBUTIONS

Mazdarani P, DVM, DVSc and Miles JE, BSc, BVetMed, PhD: Contributed to study conception and design. Material preparation and data collection were performed by all authors. Analysis was performed by Miles JE, BSc, BVetMed, PhD. The first draft of the manuscript was written by Mazdarani P, DVM, DVSc and all authors commented on previous versions of the manuscript. All authors read and approved the final manuscript.

## FUNDING INFORMATION

Institutional funding was received. The authors declare that no other funds, grants or other support were received during the preparation of this manuscript. No involvement in study design, data collection, analysis or interpretation, writing or submission occurred.

## CONFLICT OF INTEREST STATEMENT

The authors declare there are no conflicts of interest.

## Supporting information


**Data S1.** Construction details for the limb press.

**Figure S1.** Alternative data presentation to Figure 3 in the manuscript.

**Table S1.** Load cell specifications for the DYMH‐103 load cells used in this study.

**Table S2.** Main and interaction effects for the mixed‐model repeated‐measures ANOVA for the primary outcome variables.

**Table S3.** Main and interaction effects for the mixed‐model repeated‐measures ANOVA for femoral and joint angulations.

**Table S4.** Main and interaction effects for the mixed‐model repeated‐measures ANOVA for relative foot position for models 2 and 3 only.

## Data Availability

Data from this study is available online through Figshare (https://doi.org/10.6084/m9.figshare.24058686.v1).
