# Supplementary material for: Proximal femoral fixation method and axial load affect simulated muscle forces in an ex vivo feline limb press
Source: Vet Surg. 2025 Apr 7;54(5):998–1008. doi: 10.1111/vsu.14252 (PMC12282046; doi:10.1111/vsu.14252)

**Supplementary File 1: Construction details for the limb press**

*Limb press*

A custom limb press inspired by previous reports (Bilmont et al., 2018; Kneifel et al., 2018; Koch et al., 2021; Lechner et al., 2020; Retournard et al., 2016) was constructed from commonly available construction materials. The base was assembled using two pieces of 19 mm medium density fibreboard and 21 x 43 mm softwood in a torsion box construction. Four vertical holes were drilled using a guide for placement of four 500 mm long and 10 mm diameter threaded rods to support a top plate consisting of 5 mm plywood with a central reinforcement of 21 x 43 mm softwood. Wingnuts and washers were placed above and below the top plate to facilitate adjustment. A digital scale with built-in spirit level was placed on the base to measure the vertically applied force.

The top plate was prepared with two holes for placement of 5 mm threaded bolts centrally aligned from left to right, two vertical holes centrally aligned from left to right for placement of 2.0/2.4 mm positive-profile external fixator pins, and two vertical holes aligned front to back for placement of threaded bolts for mounting a DCP plate (see below). This permitted various limb press configurations to be tested as detailed in the methods section. The top plate mass was 342 g.

**Top-plate design.** The top-plate was constructed to allow three different models to be tested. A – 2 mm holes for two positive-profile external fixator pins used for the hemipelvectomized intact hip joint model (model one); B – two 5 mm holes for threaded bolts used to attach external fixator clamps and a mediolateral pin for the constrained femur model (model three); C - two 5 mm holes for threaded bolts used to attach a 3.5 mm DCP plate for the fixed femur model (model two).


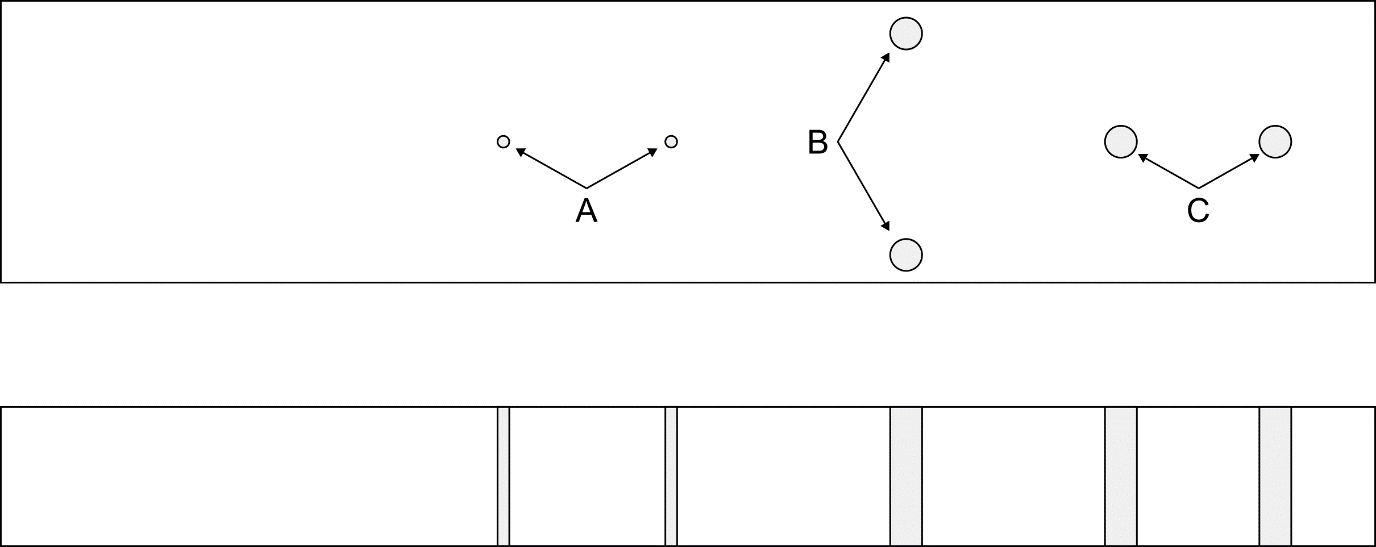

Supplement: Supplementary file 1 — Data S1. Construction details for the limb press. [file VSU-54-998-s006.docx]
