# Supplementary material for: Proximal femoral fixation method and axial load affect simulated muscle forces in an ex vivo feline limb press
Source: Vet Surg. 2025 Apr 7;54(5):998–1008. doi: 10.1111/vsu.14252 (PMC12282046; doi:10.1111/vsu.14252)

**Supplementary Figure 1:** Alternative data presentation to figure 3 in the manuscript. The same data are presented as box and whisker plots but grouped by outcome measure rather than model, at axial loads of 10% to 40% body weight. Complete – model with complete hip mobility; Rigid – model with rigidly fixed proximal femur; F-E Hip – model with flexion-extension hip mobility.


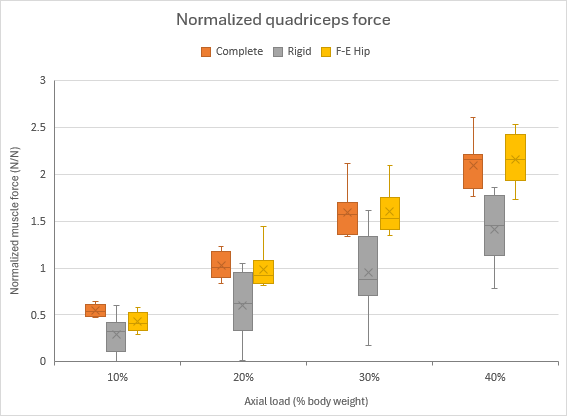


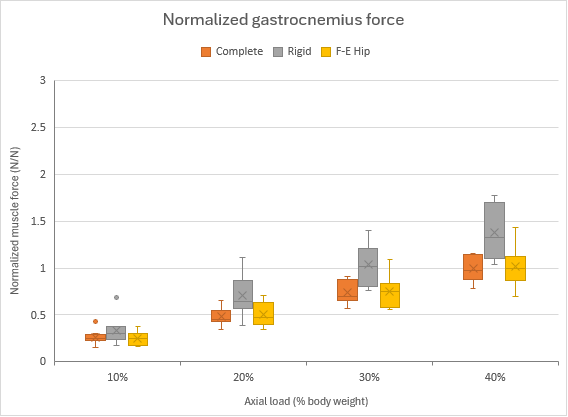


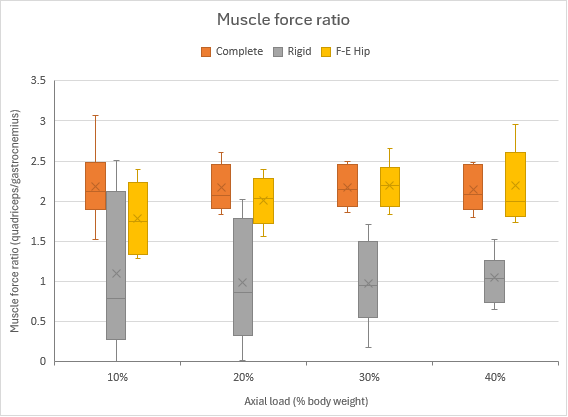

Supplement: Supplementary file 2 — Figure S1. Alternative data presentation to Figure 3 in the manuscript. [file VSU-54-998-s001.docx]
