# Supplementary material for: Proximal femoral fixation method and axial load affect simulated muscle forces in an ex vivo feline limb press
Source: Vet Surg. 2025 Apr 7;54(5):998–1008. doi: 10.1111/vsu.14252 (PMC12282046; doi:10.1111/vsu.14252)
Supplement: Supplementary file 3 — Table S1. Load cell specifications for the DYMH‐103 load cells used in this study. [file VSU-54-998-s003.docx]

**Supplementary Table 1:** Load cell specifications for the DYMH-103 load cells used in this study.

| Range | 0-10kg |
| --- | --- |
| Output sensitivity | 1.0-1.5±10%mV/V |
| Zero output | ±2% F.S |
| Nonlinear | 0.3% F.S |
| Lag | 0.03% F.S |
| Repeatability | 0.03% F.S |
| Creep (30 minutes) | 0.2% F.S |
| Temperature sensitivity drift | 0.1% F.S/10°C |
| Zero temperature drift | 0.1% F.S/10°C |
| Material | Stainless steel |
| Resistance | 350Ω |
| Insulation resistance | ≥5000MΩ/100VDC |
| Use voltage | 0-10V |
| Operating temperature range | -20~80°C |
| Safe overload | 150% |
| Extreme overload | 200% |
| Cable connection | Ex+: red; Ex-: black; Sig+: green; Sig-: white |
