# Supplementary material for: Proximal femoral fixation method and axial load affect simulated muscle forces in an ex vivo feline limb press
Source: Vet Surg. 2025 Apr 7;54(5):998–1008. doi: 10.1111/vsu.14252 (PMC12282046; doi:10.1111/vsu.14252)
Supplement: Supplementary file 4 — Table S2. Main and interaction effects for the mixed‐model repeated‐measures ANOVA for the primary outcome variables. [file VSU-54-998-s002.docx]

**Supplementary Table 2**: Main and interaction effects for the mixed-model repeated-measures ANOVA for the primary outcome variables. Both partial eta-squared and generalized eta-squared effect sizes are provided, with the former relevant for power calculations using e.g. G*Power and the latter appropriate for meta-analysis or interpretation against published effect size criteria.

| Dependent variable | Term | df | F | *P* | $\eta_{P}^{2}$ | $\eta_{G}^{2}$ |
| --- | --- | --- | --- | --- | --- | --- |
| nQ | †AxialLoad | 1.67 | 405.57 | <0.001 | 0.978 | 0.852 |
|  | †AxialLoad * Model | 3.33 | 6.67 | 0.004 | 0.597 | 0.159 |
|  | Limb | 1 | 1.75 | 0.22 | 0.163 | 0.031 |
|  | Limb * Model | 2 | 3.99 | 0.06 | 0.470 | 0.129 |
|  | †AxialLoad * Limb | 1.87 | 0.84 | 0.44 | 0.086 | 0.007 |
|  | †AxialLoad * Limb * Model | 3.75 | 2.02 | 0.14 | 0.310 | 0.033 |
|  | Model | 2 | 7.22 | 0.01 | 0.616 | 0.503 |
| nG | †AxialLoad | 1.2 | 531.01 | <0.001 | 0.983 | 0.850 |
|  | †AxialLoad * Model | 2.39 | 7.16 | 0.009 | 0.614 | 0.133 |
|  | Limb | 1 | 4.5 | 0.06 | 0.334 | 0.189 |
|  | Limb * Model | 2 | 2.53 | 0.14 | 0.359 | 0.207 |
|  | †AxialLoad * Limb | 1.33 | 2.65 | 0.12 | 0.227 | 0.022 |
|  | †AxialLoad * Limb * Model | 2.66 | 0.68 | 0.57 | 0.131 | 0.012 |
|  | Model | 2 | 9.14 | 0.007 | 0.67 | 0.423 |
| R | †AxialLoad | 1.57 | 0.83 | 0.40 | 0.084 | 0.019 |
|  | †AxialLoad * Model | 3.14 | 1.81 | 0.19 | 0.286 | 0.078 |
|  | Limb | 1 | 13.57 | 0.005 | 0.601 | 0.207 |
|  | Limb * Model | 2 | 18.48 | <0.001 | 0.804 | 0.416 |
|  | †AxialLoad * Limb | 1.72 | 0.52 | 0.58 | 0.054 | 0.007 |
|  | †AxialLoad * Limb * Model | 3.44 | 5.23 | 0.009 | 0.537 | 0.121 |
|  | Model | 2 | 23.64 | <0.001 | 0.840 | 0.723 |

df – degrees of freedom; F – F-statistic; *P* – significance level; $\eta_{P}^{2}$ – partial eta-squared effect size; $\eta_{G}^{2}$ – generalized eta-squared effect size; nQ – body weight normalized quadriceps force; nG – body weight normalized gastrocnemius force; R – nQ/nG ratio; † - Greenhouse-Geisser corrected values reported.

Pairwise comparisons between models one, two and three for the interaction (axial load*model) for body weight normalized quadriceps force (nQ). Mean differences are based on estimated marginal means and are presented along with 95% confidence intervals (CI) for the difference and associated Bonferroni-corrected *P* values.

| Axial load | Comparison | Mean difference (95% CI) | *P* |
| --- | --- | --- | --- |
|  | 1 vs. 2 | 0.26 (0.02; 0.50) | 0.037 |
| 10% | 1 vs. 3 | 0.12 (-0.13; 0.36) | 0.568 |
|  | 2 vs. 3 | -0.14 (-0.38; 0.10) | 0.375 |
|  | 1 vs. 2 | 0.43 (-0.01; 0.86) | 0.053 |
| 20% | 1 vs. 3 | 0.05 (-0.39; 0.48) | >0.99 |
|  | 2 vs. 3 | -0.38 (-0.82; 0.05) | 0.087 |
|  | 1 vs. 2 | 0.64 (0.05; 1.22) | 0.034 |
| 30% | 1 vs. 3 | -0.01 (-0.60; 0.58) | >0.99 |
|  | 2 vs. 3 | -0.65 (-1.23; -0.06) | 0.031 |
|  | 1 vs. 2 | 0.68 (0.11; 1.26) | 0.020 |
| 40% | 1 vs. 3 | -0.06 (-0.64; 0.51) | >0.99 |
|  | 2 vs. 3 | -0.75 (-1.32; -0.18) | 0.012 |

Pairwise comparisons between models one, two and three for the interaction (axial load*model) for body weight normalized gastrocnemius force (nG). Mean differences are based on estimated marginal means and are presented along with 95% confidence intervals (CI) for the difference and associated Bonferroni-corrected *P* values.

| Axial load | Comparison | Mean difference (95% CI) | *P* |
| --- | --- | --- | --- |
|  | 1 vs. 2 | -0.08 (-0.20; 0.05) | 0.323 |
| 10% | 1 vs. 3 | 0.01 (-0.11; 0.13) | >0.99 |
|  | 2 vs. 3 | 0.09 (-0.04; 0.21) | 0.21 |
|  | 1 vs. 2 | -0.22 (-0.40; -0.04) | 0.018 |
| 20% | 1 vs. 3 | -0.02 (-0.20; 0.16) | >0.99 |
|  | 2 vs. 3 | 0.20 (0.02; 0.38) | 0.03 |
|  | 1 vs. 2 | -0.30 (-0.49; -0.10) | 0.005 |
| 30% | 1 vs. 3 | -0.01 (-0.20; 0.19) | >0.99 |
|  | 2 vs. 3 | 0.29 (0.10; 0.48) | 0.005 |
|  | 1 vs. 2 | -0.39 (-0.70; -0.08) | 0.016 |
| 40% | 1 vs. 3 | -0.03 (-0.34; 0.29) | >0.99 |
|  | 2 vs. 3 | 0.36 (0.05; 0.68) | 0.023 |

Pairwise comparisons between models one, two and three for the interaction (axial load*model) for the ratio (R) of quadriceps and gastrocnemius forces. Mean differences are based on estimated marginal means and are presented along with 95% confidence intervals (CI) for the difference and associated Bonferroni-corrected *P* values.

| Axial load | Comparison | Mean difference (95% CI) | *P* |
| --- | --- | --- | --- |
|  | 1 vs. 2 | 1.10 (0.22; 1.97) | 0.016 |
| 10% | 1 vs. 3 | 0.41 (-0.47; 1.28) | 0.624 |
|  | 2 vs. 3 | -0.69 (-1.57; 0.19) | 0.138 |
|  | 1 vs. 2 | 1.18 (0.65; 1.71) | <.001 |
| 20% | 1 vs. 3 | 0.16 (-0.37; 0.69) | >0.99 |
|  | 2 vs. 3 | -1.02 (-1.55; -0.49) | <.001 |
|  | 1 vs. 2 | 1.20 (0.65; 1.75) | <.001 |
| 30% | 1 vs. 3 | -0.03 (-0.58; 0.52) | >0.99 |
|  | 2 vs. 3 | -1.22 (-1.78; -0.67) | <.001 |
|  | 1 vs. 2 | 1.09 (0.57; 1.62) | <.001 |
| 40% | 1 vs. 3 | -0.05 (-0.58; 0.48) | >0.99 |
|  | 2 vs. 3 | -1.14 (-1.67; -0.62) | <.001 |

Pairwise comparisons between left and right limbs at each axial load for models one, two and three for the ratio (R) of quadriceps and gastrocnemius forces. Mean differences are based on estimated marginal means and are presented along with 95% confidence intervals (CI) for the difference and associated Bonferroni-corrected *P* values.

| Model | Axial load | Comparison | Mean difference (95% CI) | *P* |
| --- | --- | --- | --- | --- |
| 1 | 10% | L-R | -0.47 (-1.08; 0.15) | 0.123 |
|  | 20% | L-R | -0.45 (-0.88; -0.02) | 0.041 |
|  | 30% | L-R | -0.49 (-0.79; -0.18) | 0.006 |
|  | 40% | L-R | -0.53 (-0.90; -0.15) | 0.011 |
| 2 | 10% | L-R | -1.33 (-1.95; -0.71) | <.001 |
|  | 20% | L-R | -1.11 (-1.54; -0.68) | <.001 |
|  | 30% | L-R | -0.70 (-1.00; -0.39) | <.001 |
|  | 40% | L-R | -0.41 (-0.79; -0.04) | 0.035 |
| 3 | 10% | L-R | 0.72 (0.10; 1.33) | 0.028 |
|  | 20% | L-R | 0.47 (0.04; 0.90) | 0.036 |
|  | 30% | L-R | 0.17 (-0.14; 0.47) | 0.250 |
|  | 40% | L-R | 0.23 (-0.14; 0.61) | 0.194 |
