# Supplementary material for: Proximal femoral fixation method and axial load affect simulated muscle forces in an ex vivo feline limb press
Source: Vet Surg. 2025 Apr 7;54(5):998–1008. doi: 10.1111/vsu.14252 (PMC12282046; doi:10.1111/vsu.14252)
Supplement: Supplementary file 5 — Table S3. Main and interaction effects for the mixed‐model repeated‐measures ANOVA for femoral and joint angulations. [file VSU-54-998-s005.docx]

**Supplementary Table 3**: Main and interaction effects for the mixed-model repeated-measures ANOVA for femoral and joint angulations. Both partial eta-squared and generalized eta-squared effect sizes are provided, with the former relevant for power calculations using e.g. G*Power and the latter appropriate for meta-analysis or interpretation against published effect size criteria.

| Dependent variable | Term | df | F | *P* | $\eta_{P}^{2}$ | $\eta_{G}^{2}$ |
| --- | --- | --- | --- | --- | --- | --- |
| Femur | VerticalLoad | 3 | 2.45 | 0.10 | 0.29 | 0.084 |
|  | VerticalLoad * Model | 3 | 0.89 | 0.47 | 0.129 | 0.032 |
|  | LimbSide | 1 | 3.94 | 0.09 | 0.396 | 0.123 |
|  | LimbSide * Model | 1 | 0.04 | 0.84 | 0.007 | 0.002 |
|  | VerticalLoad * LimbSide | 3 | 0.63 | 0.61 | 0.095 | 0.036 |
|  | VerticalLoad * LimbSide * Model | 3 | 1.86 | 0.17 | 0.237 | 0.099 |
|  | Model | 1 | 0.02 | 0.90 | 0.003 | 0.001 |
| Stifle | VerticalLoad | 3 | 10.06 | <0.001 | 0.626 | 0.298 |
|  | VerticalLoad * Model | 3 | 2.03 | 0.15 | 0.253 | 0.079 |
|  | LimbSide | 1 | 0.3 | 0.61 | 0.047 | 0.015 |
|  | LimbSide * Model | 1 | 0.23 | 0.65 | 0.036 | 0.012 |
|  | VerticalLoad * LimbSide | 3 | 0.92 | 0.45 | 0.132 | 0.037 |
|  | VerticalLoad * LimbSide * Model | 3 | 2.22 | 0.12 | 0.270 | 0.085 |
|  | Model | 1 | 1.81 | 0.23 | 0.231 | 0.053 |
| Hock | VerticalLoad | 3 | 14.21 | <0.001 | 0.703 | 0.497 |
|  | VerticalLoad * Model | 3 | 1.89 | 0.17 | 0.240 | 0.116 |
|  | LimbSide | 1 | 0.22 | 0.66 | 0.035 | 0.008 |
|  | LimbSide * Model | 1 | 0.47 | 0.52 | 0.072 | 0.016 |
|  | VerticalLoad * LimbSide | 3 | 2.33 | 0.11 | 0.279 | 0.066 |
|  | VerticalLoad * LimbSide * Model | 3 | 1.82 | 0.18 | 0.233 | 0.052 |
|  | Model | 1 | 0.28 | 0.62 | 0.044 | 0.009 |

df – degrees of freedom; F – F-statistic; *P* – significance level; $\eta_{P}^{2}$ – partial eta-squared effect size; $\eta_{G}^{2}$ – generalized eta-squared effect size.

Pairwise comparisons between axial loads of 10%, 20%, 30% and 40% for the caudal stifle joint angle. Mean differences are based on estimated marginal means and are presented along with 95% confidence intervals (CI) for the difference and associated Bonferroni-corrected *P* values.

| Comparison | Mean difference (95% CI) | *P* |
| --- | --- | --- |
| 10% vs. 20% | 2.59 (0.32; 4.86) | 0.027 |
| 10% vs. 30% | 3.15 (0.56; 5.74) | 0.02 |
| 10% vs. 40% | 3.27 (0.93; 5.61) | 0.01 |
| 20% vs. 30% | 0.56 (-2.30; 3.43) | >0.99 |
| 20% vs. 40% | 0.68 (-1.54; 2.90) | >0.99 |
| 30% vs. 40% | 0.12 (-3.23; 3.46) | >0.99 |

Pairwise comparisons between axial loads of 10%, 20%, 30% and 40% for the cranial hock joint angle. Mean differences are based on estimated marginal means and are presented along with 95% confidence intervals (CI) for the difference and associated Bonferroni-corrected *P* values.

| Comparison | Mean difference (95% CI) | *P* |
| --- | --- | --- |
| 10% vs. 20% | 4.12 (-0.48; 8.72) | 0.08 |
| 10% vs. 30% | 4.61 (1.30; 7.91) | 0.01 |
| 10% vs. 40% | 5.54 (2.64; 8.45) | 0.002 |
| 20% vs. 30% | 0.49 (-3.16; 4.14) | >0.99 |
| 20% vs. 40% | 1.43 (-2.15; 5.00) | >0.99 |
| 30% vs. 40% | 0.94 (-2.07; 3.94) | >0.99 |
