# Supplementary material for: Proximal femoral fixation method and axial load affect simulated muscle forces in an ex vivo feline limb press
Source: Vet Surg. 2025 Apr 7;54(5):998–1008. doi: 10.1111/vsu.14252 (PMC12282046; doi:10.1111/vsu.14252)
Supplement: Supplementary file 6 — Table S4. Main and interaction effects for the mixed‐model repeated‐measures ANOVA for relative foot position for models 2 and 3 only. [file VSU-54-998-s004.docx]

**Supplementary Table 4**: Main and interaction effects for the mixed-model repeated-measures ANOVA for relative foot position for models two and three only. Both partial eta-squared and generalized eta-squared effect sizes are provided, with the former relevant for power calculations using e.g. G*Power and the latter appropriate for meta-analysis or interpretation against published effect size criteria.

| Dependent variable | Term | df | F | *P* | $\eta_{P}^{2}$ | $\eta_{G}^{2}$ |
| --- | --- | --- | --- | --- | --- | --- |
| FS | VerticalLoad | 3 | 1.74 | 0.2 | 0.225 | 0.041 |
|  | VerticalLoad * Model | 3 | 0.44 | 0.73 | 0.068 | 0.012 |
|  | LimbSide | 1 | 1.6 | 0.25 | 0.211 | 0.125 |
|  | LimbSide * Model | 1 | 1.19 | 0.32 | 0.166 | 0.096 |
|  | VerticalLoad * LimbSide | 3 | 1.45 | 0.26 | 0.195 | 0.012 |
|  | VerticalLoad * LimbSide * Model | 3 | 1.9 | 0.17 | 0.24 | 0.016 |
|  | Model | 1 | 4.76 | 0.07 | 0.442 | 0.173 |
| SH | VerticalLoad | 3 | 0.84 | 0.5 | 0.123 | 0.014 |
|  | VerticalLoad * Model | 3 | 0.43 | 0.74 | 0.067 | 0.009 |
|  | LimbSide | 1 | 1.58 | 0.26 | 0.208 | 0.129 |
|  | LimbSide * Model | 1 | 1.18 | 0.32 | 0.165 | 0.099 |
|  | VerticalLoad * LimbSide | 3 | 1.18 | 0.35 | 0.164 | 0.014 |
|  | VerticalLoad * LimbSide * Model | 3 | 1.87 | 0.17 | 0.238 | 0.019 |
|  | Model | 1 | 7.53 | 0.03 | 0.557 | 0.255 |

df – degrees of freedom; F – F-statistic; *P* – significance level; $\eta_{P}^{2}$ – partial eta-squared effect size; $\eta_{G}^{2}$ – generalized eta-squared effect size; FS – relative craniocaudal foot position compared to femoral head and stifle joint; SH – relative craniocaudal foot position relative to stifle and hock joints.
